# Efficacy assessment of sustained intraperitoneal paclitaxel therapy in a murine model of ovarian cancer using bioluminescent imaging

**DOI:** 10.1038/sj.bjc.6604803

**Published:** 2008-11-25

**Authors:** V Vassileva, E H Moriyama, R De Souza, J Grant, C J Allen, B C Wilson, M Piquette-Miller

**Affiliations:** 1 Department of Pharmaceutical Sciences, Leslie Dan Faculty of Pharmacy, University of Toronto, 144 College Street, Rm. 1003, Toronto, Ontario, Canada M5S 3M2; 2 Division of Biophysics and Bioimaging, Ontario Cancer Institute, Princess Margaret Hospital, University of Toronto, 610 University Avenue, Suite 7-420, Toronto, Ontario, Canada M5G 2M9

**Keywords:** bioluminescent imaging, paclitaxel, intraperitoneal therapy, sustained chemotherapy, tumour proliferation, ovarian cancer

## Abstract

We evaluated the pre-clinical efficacy of a novel intraperitoneal (i.p.) sustained-release paclitaxel formulation (PTX_ePC_) using bioluminescent imaging (BLI) in the treatment of ovarian cancer. Human ovarian carcinoma cells stably expressing the firefly luciferase gene (SKOV3^Luc^) were injected i.p. into SCID mice. Tumour growth was evaluated during sustained or intermittent courses of i.p. treatment with paclitaxel (PTX). *In vitro* bioluminescence strongly correlated with cell survival and cytotoxicity. Bioluminescent imaging detected tumours before their macroscopic appearance and strongly correlated with tumour weight and survival. As compared with intermittent therapy with Taxol®, sustained PTX_ePC_ therapy resulted in significant reduction of tumour proliferation, weight and BLI signal intensity, enhanced apoptosis and increased survival times. Our results demonstrate that BLI is a useful tool in the pre-clinical evaluation of therapeutic interventions for ovarian cancer. Moreover, these results provide evidence of enhanced therapeutic efficacy with the sustained PTX_ePC_ implant system, which could potentially translate into successful clinical outcomes.

Ovarian cancer accounts for the majority of deaths from gynaecological malignancies and remains the fifth most common cancer among women (Ozols, 2003). Ovarian cancer is generally clinically silent, and therefore at the time of diagnosis the majority of patients present with advanced peritoneal metastatic disease (Bookman and Ozols, 1996; Trimble, 2006). Although a high proportion of patients attain complete clinical remission following initial treatment, unfortunately most relapse. The lack of early detection, metastasis and resistance to chemotherapy make it one of the most difficult malignancies to diagnose and treat, leaving patients with very poor prognosis.

As ovarian cancer is predominantly confined to the peritoneal cavity, strategies such as localised and sustained chemotherapy may have the potential to improve the outcome of chemotherapy. Indeed, several clinical trials have demonstrated survival advantages with localised intraperitoneal (i.p.) chemotherapy and the National Cancer Institute recommends surgery followed by combination of intravenous and i.p. chemotherapy (Ozols *et al*, 1999; Polyzos *et al*, 1999; Markman, 2001; Ozols, 2003; Rothenberg *et al*, 2003; Armstrong *et al*, 2006; Walker *et al*, 2006). Unfortunately, the majority of patients fail to complete i.p. therapy because of catheter-related complications. Therefore more tolerable therapeutic interventions are required and in this context, we developed a novel implantable paclitaxel drug delivery system (PTX_ePC_). Our initial studies demonstrated that PTX_ePC_ provided local and controlled release of PTX over several weeks and that it was safer and better tolerated than commercially available PTX formulated in Cremophor EL (Grant *et al*, 2005; Ho *et al*, 2005; Vassileva *et al*, 2007).

The availability of an intraperitoneal drug delivery system could therefore allow for further exploitation of the benefits of intraperitoneal chemotherapy while also solving the difficulties associated with the catheter system. We therefore, utilised our formulation and bioluminescent imaging (BLI) to determine whether there are therapeutic advantages in providing sustained *vs* intermittent intraperitoneal chemotherapy in ovarian cancer.

Establishment of a reliable ovarian cancer xenograft model is an important step in the pre-clinical evaluation of potential treatments. However, monitoring the development and progression of peritoneal disease is difficult. Traditional assessments of efficacy are based on measuring total tumour mass, including areas of necrosis and oedema and do not necessarily evaluate the effects of treatment on the number of viable tumour cells without additional processing and evaluation. In this context, animals must be killed at pre-determined end points, increasing the number of animals required and limiting the possibility of ongoing observation of tumour progression within the same animal. Recently BLI has emerged as a real-time non-invasive method to follow tumour progression (Contag *et al*, 1997; Edinger *et al*, 2003; Jenkins *et al*, 2003a, 2003b; Hollingshead *et al*, 2004; Craft *et al*, 2005). Bioluminescent imaging detects only live, metabolically active tumour cells, which is important in the monitoring of a therapeutic response (Jenkins *et al*, 2003a, 2003b; Hollingshead *et al*, 2004; Sato *et al*, 2004). Although in recent years BLI has been widely utilised, to date there are scarce reports on BLI and ovarian cancer and none in particular on sustained intraperitoneal chemotherapy. We therefore, stably transfected the SKOV3 human ovarian carcinoma cell line with the firefly luciferase gene and utilised BLI to evaluate the effects of sustained and intermittent i.p. Paclitaxel chemotherapy on ovarian tumour response.

## Materials and methods

### Tumour cell line co-transfection and selection

The SKOV3 human ovarian adenocarcinoma cell line was obtained from the American Type Culture Collection (Rockville, MD, USA). Cells were grown in monolayer cultures in RPMI-1640 medium supplemented with 10% fetal bovine serum and 1% penicillin/streptomycin (Invitrogen, Burlington, ON, Canada) in a humidified atmosphere of 5% CO_2_. SKOV3 cells were stably co-transfected with the modified firefly luciferase gene plasmid, pGL3 enhancer vector and the pCI-neo mammalian expression vector for neomycin selection (Promega, Madison, WI, USA) with the Fugene-6 transfection kit (Roche, Laval, QC, Canada). Following transfection, cells were cultured in G418 sulfate (800 *μ*g ml^−1^) containing medium for 10 days. Surviving colonies were exposed to 1 mM D-luciferin potassium salt (Promega, Madison, WI, USA) and imaged by IVIS™ Imaging System (Xenogen, Alameda, CA, USA) for selection of stably co-transfected luciferase/neo clones (SKOV3^Luc^). Following isolation, SKOV3^Luc^ cells were maintained and propagated in 400 *μ*g ml^−1^ G418 sulfate-containing medium. SKOV3^Luc^ cells were imaged each week to ensure sustained *luciferase* expression and studies were commenced 2 months post transfection. Furthermore, to evaluate the stable expression of *luciferase* over time, SKOV3^Luc^ cells were continuously cultured in G418 sulfate-free media for 3 months and were periodically imaged. *Luciferase* expression remained stable relative to the initial imaging results.

### PTX cytotoxicity in SKOV3^Luc^ cells

Exponentially growing SKOV3^Luc^ cells were exposed to various PTX concentrations ranging from 0 to 17 080 nM for 24, 48 and 72 h. Cells were then exposed to D-luciferin potassium salt, 1 mM in phosphate-buffered saline (Promega, Madison, WI, USA) and imaged to determine bioluminescence as described below. Cell viability was determined by the MTT [3-(4,5-dimethylthiazol-2-yl)-2,5-di.p.henyl tetrazolium bromide] assay as described earlier (Mosmann, 1983; Ho *et al*, 2005) and the % cell survival was determined as: ((absorbance_(*λ*=560 nm)_ treated ÷ absorbance _(*λ*=560 nm) untreated_) × 100). Linear regression analysis was performed to determine whether a good correlation exists between bioluminescent signal ((Bioluminescence_treated_ ÷ Bioluminescence signal_untreated_) × 100) and cell survival in treated SKOV3^Luc^ cells. Experiments were performed in triplicate.

### Bioluminescent imaging

Bioluminescent imaging was performed with the IVIS™ Imaging System, composed of a high sensitivity cooled charge-coupled camera mounted in a light-tight box (Xenogen, Alameda, CA, USA). The commercially available Living Image™ software (Xenogen, Alameda, CA, USA) was used to collect and analyse images. For *in vitro* imaging, the bioluminescent signal was obtained over a 60-s integration period. For *in vivo* imaging, mice were pre-anaesthetised (Ketamine/Xylazine, 100, 10 mg kg^−1^ respectively) and were administered D-luciferin potassium salt in saline (100 mg kg^−1^) intraperitoneally. Animals were then placed in an air-tight box in the imaging chamber (with heated stage, 37°C) at a distance allowing for whole body imaging in a single field of view. Gray-scale images were initially obtained to provide an anatomical reference for the bioluminescent images, which were then acquired over a 5-min integration time. Regions of interest covering the entire peritoneal cavity were selected, including tumours and total photon counts were determined. Bioluminescent images were collected at various time points throughout the duration of the study.

### Generation of xenografts

Female SCID mice (4- to 6-week-old, 18–20 g), obtained from the University Health Network animal colony (Toronto, ON, Canada) were injected intraperitoneally with 1 × 10^7^ SKOV3^Luc^ cells, suspended in 200 *μ*l of RPMI-1640 medium. Animals were housed under sterile conditions in microisolator cages, fed standard chow diet with water *ad libitum* and maintained on an automatic 12-h light cycle at 22–24°C. All studies were conducted using sterile techniques and in accordance with the guidelines of the University Health Network Animal Care Committee and the Canadian Animal Care Council.

### Treatment groups

Tumour bearing mice received PTX (60 mg kg^−1^ total dose over 3 weeks) either as intermittent therapy (i.p. bolus injections of 20 mg kg^−1^ Taxol® on a q7d × 3 schedule) or sustained therapy (PTX_ePC_ surgically implanted i.p., providing sustained delivery of 20 mg kg^−1^ per week) and were compared with controls (drug-free ePC implant). Prior to treatment initiation, release from PTX_ePC_ was confirmed both *in vitro* and *in vivo* as described earlier (Grant *et al*, 2005; Ho *et al*, 2005; Vassileva *et al*, 2007). Treatment was initiated 7 days post SKOV3^Luc^ inoculation, referred to as Day 0; on this date, three mice were killed for visual and microscopic tumour inspection/analysis, and the remainder of the animals (*n*=36), were separated into the various groups as described above (*n*=12/each group). Mice were imaged every 2 days post SKOV3^Luc^ tumour inoculation, at treatment initiation, 24-h post-treatment initiation and every 2–3 days throughout the course of treatment until the termination of the study. Each week (Days 7, 14 and 21), three mice from each group were randomly selected and killed for analysis of tumours and tissues. Owing to rapid disease progression, a maximum 4-week monitoring period was established. Animals were monitored daily for normal activity, abdominal swelling (peritonitis), weight loss, muscle wasting, abdominal distension, and under-conditioning to assess treatment-related toxicities and disease progression. To monitor abdominal distension, we utilised an autoclavable, fabric measuring tape to measure abdominal girth two times weekly, commencing at the day of tumour inoculation. To assess under-conditioning and muscle wasting, we used the ‘body conditioning scoring’ system as described earlier (Ullman-Culleré and Foltz, 1999). Briefly, this was done by monitoring changes in flesh coverage of bony protuberances. This has been shown to accurately reflect the health decline in mice that have organ enlargement, such as tumour burden growth. End points included: (1) inactivity, hypothermia, or hunched posture; (2) muscle wasting and under-conditioning using body condition scoring; (3) weight loss or gain in excess of 20%; (4) ulceration/infection at tumour site; or (5) excessive distension of the abdomen as assessed by abdominal girth measurements. Animals that reached these pre-determined ethical end points related to tumour burden and body conditionings were killed. Kaplan–Meier survival curves were constructed.

Plasma, tumour and liver drug concentrations were measured by high performance liquid chromatography as described earlier (Vassileva *et al*, 2007). As intraperitoneal ovarian xenografts disseminate throughout the peritoneal cavity, all macroscopic (visible) tumour nodules were excised, harvested and weighed, followed by fixation in 4% paraformaldehyde (PFA) solution. Tumour tissues were paraffin embedded, processed and sectioned. Markers of proliferation (Ki-67) and apoptosis (terminal deoxytransferase-mediated dUTP nick-end labeling and Caspase 3) were examined.

### Immunohistochemistry

Paraffin sections were dewaxed in xylene, passaged through graded alcohols and rinsed in distilled water. Sections were immersed in 10 mM citrate buffer, pH 6, at 95–100°C for 20 min in a microwavable pressure cooker; cooled and rinsed in PBS, then treated with 1% Pepsin (Sigma, Oakville, ON, Canada) in 0.01 N HCl (pH 2.0) for 15 min at 37°C. Endogenous peroxidase and biotin activity were blocked using 3% hydrogen peroxide and avidin/biotin blocking kit (Lab Vision, Fremont, CA, USA). For the assessment of proliferation, sections were incubated at room temperature with the Ki-67 monoclonal antibody (Invitrogen, Burlington, ON, Canada), 1/50 dilution for 1 h. For the assessment of apoptosis, sections were incubated with active caspase 3 (casp3) antibody (Chemicon, Temecula, CA, USA), 1/50 dilution overnight, or with biotin-nucleotide cocktail and DNA polymerase 1 (Promega, Madison WI, USA) for 1 h at 37°C for *in situ* terminal deoxytransferase-mediated dUTP nick-end labeling (TUNEL). Colour development was done with freshly prepared NovaRed solution (Vector Laboratories, Burlington, ON, Canada). Sections were counterstained lightly with Mayer's haematoxylin, dehydrated in alcohols, cleared in xylene and mounted in Permount (Fischer, Ottawa, ON, Canada). Nuclei that stained brownish-red were scored as positive and those that stained blue were scored as negative.

### Quantification of tumour proliferation and apoptosis

Tumour sections were imaged using a Nikon Coolpix 990 colour camera mounted on a Nikon Eclipse e400 microscope (Japan). At least 10 fields ( × 400) were randomly selected from each slide. Positively and negatively stained nuclear areas were collected by using Image J analysis software (NIH, Bethesda, MD, USA). Ki-67, Casp3 and TUNEL-labeling indices were determined as the ratio of areas occupied by the positively stained tumour cell nuclei relative to all tumour cell nuclei.

### Statistical analysis

Data are expressed as means±s.e. Data were analysed using one-way ANOVA and the paired Student's *t*-test of unequal variance for comparison between groups. Regression analysis was performed to establish correlations and the log-rank test for survival analysis. Differences were considered statistically significant at *P*<0.05.

## Results

### *In vitro* evaluation of PTX in SKOV3^Luc^ cells

**Figure 1:**
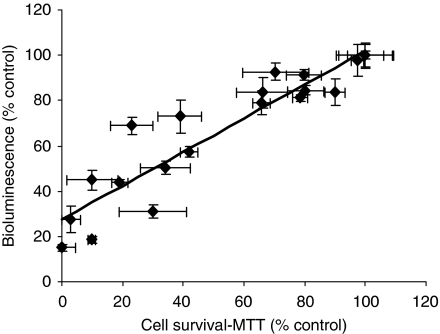
Correlation between bioluminescence and the MTT cell viability assay in SKOV3^Luc^ cells in response to various PTX concentrations and exposure times. There was a significant correlation between both assays (*r*=0.93, *P*<0.05). Experiments were conducted in triplicates. Data presented as mean±s.e.

PTX inhibited SKOV3^Luc^ cell survival in a concentration and time-dependent manner as evaluated by BLI and MTT. As duration of exposure was increased, cell survival decreased and the IC_50_ values were 683±72, 137±29 and 27±12 nM PTX at 24, 48 and 72 h, respectively. The bioluminescent signal (% control) significantly correlated with the MTT assessment of cell viability (Figure 1). Furthermore, SKOV3^Luc^ sensitivity to PTX was similar to the parental SKOV3 cell line (data not shown).

### *In vivo* evaluation of sustained and intermittent PTX chemotherapy

**Figure 2:**
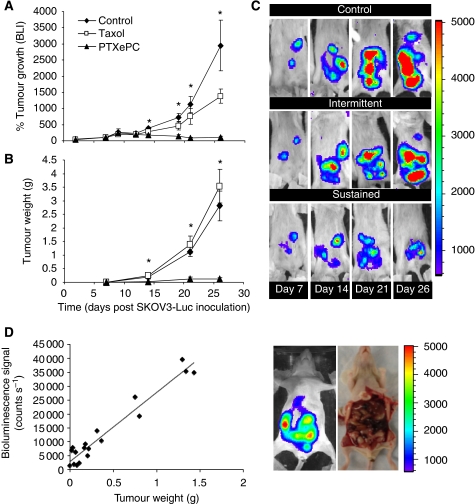
Tumour growth of SKOV3^Luc^ xenografts in response to intraperitoneal chemotherapy. Paclitaxel therapy (60 mg kg^−1^ total over 3 weeks) was initiated 7 days post SKOV3^Luc^ inoculation. Intermittent therapy consisted of 20 mg kg^−1^ on q7d × 3 schedule (days 7, 14, 21) and sustained therapy consisted of surgical implantation of PTX_ePC_ releasing 20 mg kg^−1^ per week (*n*=9 per group). Non-treated and drug-free ePC implant controls were combined (*n*=18). (**A**) Relative bioluminescence – bioluminescent signal (counts/s) was normalised to treatment initiation date for individual animals (day 7 post SKOV3^Luc^ inoculation); the number of mice per group changed as the study proceeded, therefore the bioluminescent signal was obtained from *n*=39 on day 0 and 7; *n*=6–15 per group on day 14; *n*=3–8 per group on day 21 and *n*=4–5 per group on day 26; (**B**) Tumour growth curve; *n*=3–6 tumours per group at each time point (obtained from *n*=3–6 mice per group); (**C**) Representative examples of successive *in vivo* bioluminescent images of tumour growth in the same animal for each group. Each image was collected for 5 min. The colour scale bar indicates photon count per pixel. There was a significant difference in tumour growth between groups as measured by bioluminescence and tumour weight, *P*<0.05, ANOVA (^*^). (**D**) Regression analysis revealed significant correlation between bioluminescence and tumour weight (*r*=0.97, *P*<0.05). The anatomical location of tumours upon macroscopic examination reflected bioluminescence.

**Figure 3:**
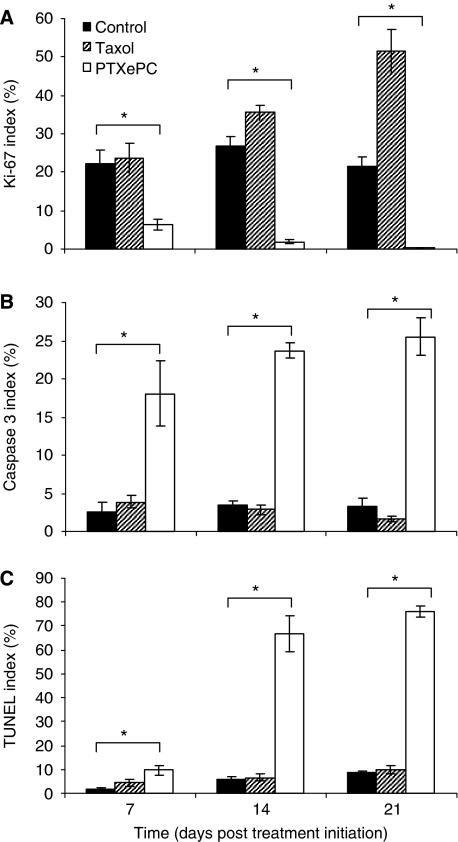
Effect of intermittent Taxol and sustained PTX_ePC_ therapy on tumour proliferation and apoptosis in SKOV3^Luc^ xenografts. (**A**) Ki-67, (**B**) Caspase 3 and (**C**) TUNEL indices. There was a significant difference in tumour proliferation and apoptosis between all groups at each time point, *P*<0.05, ANOVA (^*^); *n*=3–6 tumours per group, with at least 10 fields per tumour randomly selected from each slide. Data presented as mean±s.e.

**Figure 4:**
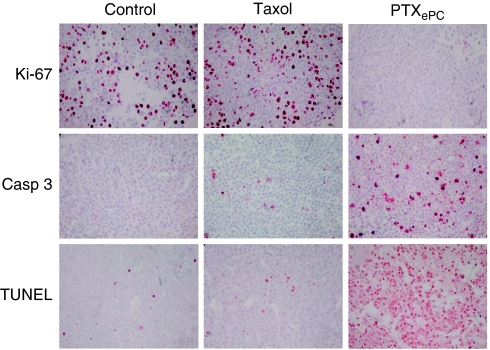
Representative tumour sections displaying the effect of intermittent Taxol and sustained PTX_ePC_ therapy on tumour proliferation and apoptosis in SKOV3^Luc^ xenografts. Paraffin sections, 5 *μ*m and × 400 magnification. Nuclei stained red/brown were scored as positive and nuclei that stained blue were scored as negative. Sections obtained on day 14 post-treatment initiation.

The effect of sustained *vs* intermittent intraperitoneal PTX administration was examined *in vivo* in the SKOV3^Luc^ xenograft model of human ovarian cancer in SCID mice. Sustained intraperitoneal PTX delivery with PTX_ePC_ resulted in significant reduction of tumour growth, whereas intermittent therapy with Taxol had no significant effect on tumour reduction as measured by bioluminescent imaging and tumour weight (Figure 2A–C). Bioluminescence counts strongly correlated with tumour weight (Figure 2D). The difference in tumour growth between treatment groups was also reflected by Ki-67 proliferation indices. Sustained PTX administration with PTX_ePC_ significantly reduced the proliferation index of tumours as compared with both control- and Taxol-treated mice. Moreover, intermittent therapy with Taxol resulted in a significant gradual increase in tumour proliferation as compared with control- and PTX_ePC_-treated animals (Figures 3A and 4). Proliferation indices significantly correlated with tumour weight (*r*=0.98, *P*<0.05) and bioluminescence signal (*r*=0.99, *P*<0.05).

**Figure 5:**
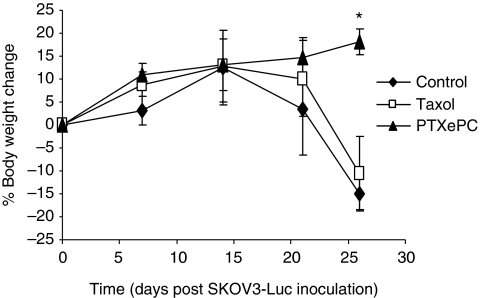
Body weight changes in response to chemotherapy. Control- and Taxol-treated animals encountered significant weight loss by day 25, indicating disease progression. Furthermore, weights in the PTX_ePC_ treatment group significantly differed from control- and Taxol-treated animals, *P*<0.05, Student's *t*-test (^*^). The number of mice per group changed as the study proceeded, therefore body weights were obtained from *n*=39 on day 0 and 7; *n*=6–15 per group on day 14; *n*=3–8 per group on day 21 and n=4–5 per group on day 26. Data presented as mean±s.e.

To further characterise tumour response to treatment, we investigated the degree of apoptosis as measured by Caspase 3 and TUNEL. Sustained PTX_ePC_ treatment significantly increased the proportion of apoptotic tumour cells, whereas intermittent Taxol therapy had no effect (Figures 3B and C and 4). Moreover, significant decreases in body weight of control- and Taxol-treated animals was observed by day 25 post SKOV3^Luc^ inoculation, whereas sustained therapy had no deleterious effects (Figure 5), suggesting that PTX_ePC_ chemotherapy was more effective and better tolerated.

### Effects of PTX chemotherapy on survival

**Figure 6:**
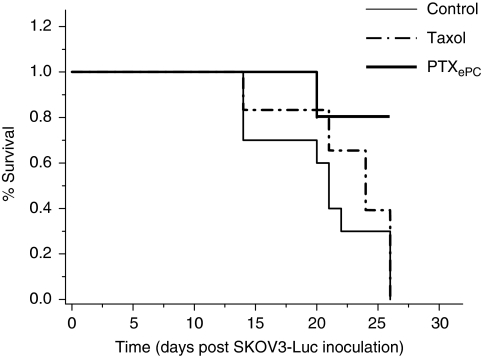
The effects of intermittent Taxol and sustained PTX_ePC_ chemotherapy on survival (Kaplan–Meier curve). Taxol therapy did not result in significant survival advantages in comparison to controls (*P*=0.17), whereas sustained PTX_ePC_ therapy resulted in significant survival advantages in comparison to control (*P*=0.0001) and Taxol-treated (*P*=0.0002) animals.

Sustained PTX administration with PTX_ePC_ significantly increased the probability of survival, whereas intermittent Taxol treatment had no significant effects on survival probability (Figure 6). The median survival times (post-tumour inoculation) for the control and intermittent (Taxol) group were 21 and 25 days, respectively. In the PTX_ePC_ group, the probability of survival was 89%, however, all animals were killed on day 26, therefore long-term survival rates, could not be determined.

### PTX concentration levels

PTX concentration levels were measured in plasma, liver, tumour and ascites each week. In the PTX_ePC_ group, sustained PTX plasma concentrations were seen from days 7 to 21 of treatment. Paclitaxel concentration levels from the PTX_ePC_-treated group are displayed in Table 1. As samples were collected 1 week following the final dose of Taxol, it was not surprising that PTX levels were below detection limits in all plasma and tissue samples of the intermittent treatment group.

## Discussion

Although intraperitoneal tumour models may offer close representation of clinical disease progression, the assessment of therapeutic efficacy has proven to be a difficult and daunting task because of animal-to-animal variability. In this context, we created a stably luciferase-expressing ovarian cancer cell line and developed an intraperitoneal xenograft model that closely mimics metastatic ovarian cancer. We observed strong correlations between the bioluminescent signal with tumour weight and cell viability and thus employed bioluminescent imaging as a tool to investigate the effects of sustained *vs* intermittent intraperitoneal administration of PTX on ovarian tumour responsiveness. There are a number of BLI studies monitoring tumour response to different treatments (Contag *et al*, 1997; Jenkins *et al*, 2003b; McCaffrey *et al*, 2003; Hollingshead *et al*, 2004; Peter *et al*, 2007); however, our report is the first to explore the potential of sustained intraperitoneal chemotherapy with real-time progressive observation in a human ovarian cancer model.

Similarly to previous studies with other cell lines (Jenkins *et al*, 2003a, 2003b; Hollingshead *et al*, 2004), SKOV3^Luc^ cell survival strongly correlated with bioluminescence. For instance, when SKOV3^Luc^ cells were exposed to PTX for 24, 48 or 72 h, bioluminescence decreased with increasing concentration, which strongly correlated with the decrease in cell survival. Furthermore, we demonstrate that SKOV3^Luc^ cells grew rapidly *in vivo* and generated tumours that were detectable by BLI as early as 4 days post inoculation, which is otherwise impossible with traditional detection methods. Our tumour model provided sensitive and non-invasive monitoring of intraperitoneal disease and allowed us to initiate treatment when tumours reached similar sizes in all animals. This way, animals with similar-sized tumours were randomised into the various treatment groups and were monitored throughout the course of therapy. The luciferase activity in tumours showed excellent correlation to tumour weight and proliferation, thus validating the model with more traditional tumour evaluation methods. Furthermore, the anatomical location of tumours corresponded to the image obtained by BLI. Similarly, studies with other tumour models have also reported correlations between bioluminescence and tumour weight and/or volume (Peter *et al*, 2007; Wang *et al*, 2007), hence supporting the technique as a sensitive and reliable method of efficacy evaluation.

Although BLI has emerged as a powerful tool for the monitoring of *in vivo* disease progression and regression, its use in endogenous tumours is limited as it relies on the expression of a foreign gene and therefore cannot be directly translated into the clinic (Hollingshead *et al*, 2004). Furthermore, it is restricted to small research animals or to the superficial tissues of larger animals as signal intensity may be attenuated by scattering (Jenkins *et al*, 2003a). Bioluminescent imaging is two-dimensional and unable to obtain in-depth information; however, the development of bioluminescent tomography has overcome these limitations and three-dimensional images are readily generated (Kumar *et al*, 2007; Zhang *et al*, 2008). Bioluminescent imaging can therefore provide an important window into the effect of therapy.

Sustained PTX chemotherapy provided by the PTX_ePC_ implant significantly reduced tumour growth and enhanced therapeutic response and survival rates in comparison to intermittent Taxol administration at equivalent doses. For instance, tumour bioluminescence gradually increased by ∼27 and 11-fold in the tumours of the control- and Taxol-treated mice, respectively, with regard to treatment initiation date. Meanwhile, bioluminescence in the PTX_ePC_-treated mice did not intensify, indicating that sustained therapy was more effective. Furthermore, PTX_ePC_ treatment decreased tumour mass and proliferation by ∼96% and ∼63-fold, respectively, as compared with controls, whereas intermittent therapy resulted in increased tumour growth. Tumour weights and proliferation indices were ∼29 and ∼152-fold greater, respectively, in the Taxol group in comparison to PTX_ePC_. Interestingly, intermittent Taxol administration resulted in a significant gradual increase in proliferative indices in comparison to non-treated controls, suggesting possible tumour repopulation in between treatment cycles. Indeed, it is thought that intermittent chemotherapy may result in increased rates of tumour repopulation during treatment-free intervals, and thus limit efficacy (Kim and Tannock, 2005). In this context, there is extensive evidence supporting the occurrence of increased tumour repopulation rates during courses of fractionated radiotherapy; implementation of continuous low-dose radiation schedules have resulted in substantial improvements in local tumour control and survival times (Malaise and Tubiana, 1966; Szczepanski and Trott, 1975; Jung *et al*, 1990). Similarly, we have recently demonstrated that sustained Taxol chemotherapy resulted in decreased tumour proliferation and diminished tumour repopulation, as compared with intermittent therapy (Edinger *et al*, 2003).

In addition to diminished tumour repopulation, this enhanced tumour responsiveness with PTX_ePC_, may be as a result of both regional and continuous drug exposure. As ovarian tumours are predominantly confined to the peritoneal cavity, intraperitoneal administration allows for high PTX concentrations directly at the tumour site, whereas continuous exposure increases the proportion of apoptotic tumour cells (Dedrick *et al*, 1978; Rowinsky and Donehower, 1995). Indeed, in the PTX_ePC_ treatment group, PTX concentrations in ascites and tumours were >2 and >110-fold greater than in plasma, respectively, and were accompanied by extensive tumour apoptosis. For instance, the degree of apoptosis in tumours obtained from the PTX_ePC_ treatment group was >16 and 12-fold greater than Taxol and control groups, respectively, indicating that sustained therapy was more effective. Improved and more consistent tumour drug penetration have been previously demonstrated with increased exposure time to PTX, both in xenografts and in patient tumours (Kuh *et al*, 1999). Additional targeting of vascularised tumours likely occurred through the circulation as clinically relevant and tolerable drug plasma levels were achieved in the PTX_ePC_-treated group (range of 12–31 ng ml^−1^ in all animals). However, much greater concentrations were accomplished in the peritoneal cavity at the tumour site. Therefore, PTX_ePC_ therapy achieved augmented tumour targeting and diminished systemic toxicity. Alternatively, peak plasma and liver concentration levels have been reported to be about 17 *μ*g ml^−1^ and 60 *μ*g g^−1^ respectively, following a single i.p. bolus doses of PTX (20 mg kg^−1^); concentrations associated with numerous toxicities (Tamura *et al*, 1995; Glantz *et al*, 1996; Maier-Lenz *et al*, 1997; Kuzuya *et al*, 2001; Bardelmeijer *et al*, 2003). The diminished toxicity and enhanced efficacy with PTX_ePC_ was also reflected by the lack of significant changes in body weight. Indeed, we have previously demonstrated favourable toxicity profiles with PTX_ePC_ and augmented toxicity with Taxol (Vassileva *et al*, 2007). Similarly, weight loss may be reflective of efficacy, as physical wasting is an attribute of disease progression with this model. Both control- and Taxol-treated animals encountered significant weight loss by day 25, indicating disease progression and limited efficacy in the case of intermittent Taxol therapy. Therefore, intermittent therapy results in high systemic levels, which are associated with dose-limiting toxicity and a short-lived therapeutic window.

Localised chemotherapy alleviates systemic toxicities and has demonstrated survival advantages in advanced ovarian cancer patients; however, catheter-related complications currently remain a limitation in the clinical application of intraperitoneal chemotherapy. Our drug delivery system may be a solution to the problems encountered with current intraperitoneal chemotherapy administration practices and allow further exploration of localised drug delivery. Moreover, our formulation provides sustained-release of PTX, which may further improve efficacy by diminishing the rates of tumour repopulation and by enhancing tumour cell apoptosis. We also demonstrate that bioluminescent imaging enables earlier detection of tumours than traditional methods and that signal intensity correlates with tumour growth. Therefore, bioluminescent imaging is a non-invasive and rapid approach for the monitoring of experimental tumours and should be further utilised in the pre-clinical evaluation of therapeutic interventions for ovarian cancer.

## Figures and Tables

**Table 1:** Paclitaxel concentrations detected in PTX_ePC_-treated animals

**Time^a^**	**Plasma (ng ml^−1^)**	**Tumour (ng g^−1^)**	**Ascites (ng ml^−1^)**	**Liver (ng g^−1^)**
7	17±2	337±7	28±2	345±155
14	22±4	2443±139	52±7	691±400
21	24±7	1102±609	56±5	558±310

a Days post treatment initiation.

Sustained PTX concentrations were observed in the PTX_ePC_ group from days 7 to 21 of treatment. No significant differences in concentration levels were found. Concentrations based on *n*=3 per time point.
